# Protecting the Children

**Published:** 1920-04-10

**Authors:** 


THE PUBLIC WELL-BEING—(*continued*)

### Protecting the Children.

On April 1 a number of important provisions of the Education Act came into force.

No child under twelve years of age may be employed at all.

No child between twelve and fourteen may be employed for more than two hours on Sunday or on school days, except after school hours and before 8 p.m., and on other days except between 6 a.m. and 8 p.m. Local authorities may pass by-laws permitting the employment of children on a school day before 9 o'clock in the morning, provided the employment is limited to one hour, and that the child so employed shall not be employed for more than one hour in the afternoon.

The hour after which child performers are not allowed to appear is altered from 9 p.m. to 8 p.m., and the permissible age from eleven to twelve. Licences will in future be within the discretion of the local education authority.

Section 18 provides for medical inspection and treatment of pupils in secondary and continuation schools.

By Section 20 local authorities can ascertain what children in their areas are physically defective or epileptic, and the provisions of the Elementary Education Act (1914) relating to mentally defective children shall be extended to physically defective and epileptic children.

### A Health Chain.

Another important step which has been taken is the issue of regulations by the Board of Education (at the suggestion of Sir Robert Newman, Chief Medical Officer of the Ministry of Health), ensuring a medical record of all children passing from elementary to secondary schools. A child will thus have a complete medical record throughout the school life, and when the time comes for it to enter upon a career there will be a dossier of health for guidance. This continuous record is expected to be of great advantage from the standpoint of the individual, of public health, of medical observation and research, and of social reform. There have never before been such complete statistics as will now be collected. The school supervision, with ante-natal clinics, day nurseries, nursery schools, welfare centres, works welfare organisations, national health insurance, and so forth, are gradually building up a great national health chain from the cradle—even before that—to the workshop, and it is hoped that they will have a lasting effect on the health, happiness, and efficiency of coming generations.

